# STAT3 Activities and Energy Metabolism: Dangerous Liaisons

**DOI:** 10.3390/cancers6031579

**Published:** 2014-07-31

**Authors:** Annalisa Camporeale, Marco Demaria, Emanuele Monteleone, Carlotta Giorgi, Mariusz R. Wieckowski, Paolo Pinton, Valeria Poli

**Affiliations:** 1 Molecular Biotechnology Center and Department of Molecular Biotechnology and Life Sciences, University of Turin, Via Nizza 52, Turin 10126, Italy; E-Mail: emanuele.monteleone@unito.it; 2 Buck Institute for Research on Aging, 8001 Redwood Blvd, Novato, CA 94945, USA; E-Mail: mdemaria@buckinstitute.org; 3 Department of Experimental and Diagnostic Medicine, Section of General Pathology, Laboratory for Technologies of Advances Therapies (LTTA), University of Ferrara, Via Fossato di Mortara 70, Ferrara 44121, Italy; E-Mails: grgclt@unife.it (C.G.); pnp@unife.it (P.P.); 4 Nencki Institute of Experimental Biology, Department of Biochemistry, Pasteur Str. 3, Warsaw 02-093, Poland; E-Mail: m.wieckowski@nencki.gov.pl

**Keywords:** STAT3, metabolism, Warburg effect, aerobic glycolysis, cellular transformation, mitochondrial activity

## Abstract

STAT3 mediates cytokine and growth factor receptor signalling, becoming transcriptionally active upon tyrosine 705 phosphorylation (Y-P). Constitutively Y-P STAT3 is observed in many tumors that become addicted to its activity, and STAT3 transcriptional activation is required for tumor transformation downstream of several oncogenes. We have recently demonstrated that constitutively active STAT3 drives a metabolic switch towards aerobic glycolysis through the transcriptional induction of *Hif*-1α and the down-regulation of mitochondrial activity, in both MEF cells expressing constitutively active STAT3 (*Stat3^C/C^*) and STAT3-addicted tumor cells. This novel metabolic function is likely involved in mediating pre-oncogenic features in the primary *Stat3^C/C^* MEFs such as resistance to apoptosis and senescence and rapid proliferation. Moreover, it strongly contributes to the ability of primary *Stat3^C/C^* MEFs to undergo malignant transformation upon spontaneous immortalization, a feature that may explain the well known causative link between STAT3 constitutive activity and tumor transformation under chronic inflammatory conditions. Taken together with the recently uncovered role of STAT3 in regulating energy metabolism from within the mitochondrion when phosphorylated on Ser 727, these data place STAT3 at the center of a hub regulating energy metabolism under different conditions, in most cases promoting cell survival, proliferation and malignant transformation even though with distinct mechanisms.

## 1. Introduction

The transcription factor Signal Transducer and Activator of Transcription 3 (STAT3) is activated downstream of many cytokine and growth factor receptors, resulting in tyrosine phosphorylation, dimerization via reciprocal phosphotyrosine-src homology (SH)-2 interactions and translocation to the nucleus, where it binds to responsive elements on gene promoters. STAT3 can also be phosphorylated on serine residue 727 (S-P) within its carboxyl-terminal Transcription Activation Domain [1,2], which is thought to provide full trans-activating properties to the Y-P protein for optimal induction of a subset of target genes [3].

STAT3 target genes and functions vary depending on the cellular system, but in most instances correlate with cell survival and proliferation. Under physiological conditions, STAT3 activation is transient and tightly regulated by negative feedback mechanisms mediated, among others, by suppressor of cytokine signal (SOCS) and protein inhibitor of activated STAT (PIAS) proteins [4,5]. On the other hand, persistent STAT3 activation is commonly observed in tumors of different origin and during chronic inflammation, downstream of continuous cytokine/growth factor stimulation or of constitutive activity of non-receptor tyrosine kinases such as cSrc [6]. A direct role in oncogenesis was demonstrated by the ability of the artificially mutated form *Stat3C* to lead to malignant transformation when overexpressed in immortalized fibroblasts and epithelial cells [7]. STAT3 inhibition in many tumor models/primary tumor cells leads to loss of survival and proliferation, suggesting addiction of tumor cells to STAT3 activity [8,9]. Accordingly, STAT3 is required for malignant transformation by many oncogenes, in primis vSrc, in a number of cell types [10].

In tumors, STAT3 is known to exert a number of well established functions correlating to transcriptional activation of its target genes, including regulation of cell-cycle progression, apoptosis, tumor angiogenesis, invasion, metastasis, and tumor cell evasion from the immune system, reflecting the involvement of this factor in multiple steps of the oncogenic program [11]. Additionally, STAT3 is considered as a key player in mediating inflammation-driven tumorigenesis, being constitutively activated by chronically high levels of the pro-inflammatory cytokine IL-6 [12].

### 1.1. STAT3 Differentially Modified Forms and Cell Metabolism

Aberrant regulation of cell metabolism plays a central role in deterring the survival and growth features of malignant cells. For example, most tumor cells display a metabolic switch towards aerobic glycolysis, with increased glycolysis and decreased oxidative phosphorylation, even under conditions of high oxygen tension [13,14,15]. This phenomenon is thought to favor the synthesis of essential cellular components required for fast cell duplication. Recently, a number of observations have suggested that STAT3 can act as a central regulator of cell metabolism at multiple levels, which may represent a core function in promoting growth/survival of biologically distinct tumors [16]. Interestingly, specific and often unconventional functions have been assigned to the differentially modified forms of this factor, as summarized below.

First, STAT3 was shown to localize to mitochondria, where its S-P form enhances coupled Complex I and II activity and reduces ROS production, while inducing aerobic glycolysis [17,18]. This function enhances cell survival under stress conditions such as heart ischemia, and is required for cell transformation downstream of Ras oncogenes, which elicit S, but not Y, phosphorylation of STAT3. Additionally, STAT3 was shown to interact with cyclophilin F (best known as cyclophilin D) in the mitochondrial matrix, thus inhibiting the opening of the mitochondrial permeability transition pore (MPTP) and Calcium-induced apoptosis [19]. STAT3 S-P is known to enhance its interaction with the complex I component GRIM-19, responsible for its mitochondrial translocation [20]. Although how STAT3 phosphorylation can be regulated within the mitochondrion is not understood, the phosphatase SHP2 was proposed as a potential player in dephosphorylating mitochondrial STAT3 [21].

Y-P STAT3 was also recently shown to play important roles in regulating energy metabolism. Indeed, we have demonstrated that constitutively Y-P STAT3 can promote aerobic glycolysis and down-regulate mitochondrial activity, partly acting via transcriptional induction of its well-recognized transcriptional target *Hif*-1α [22]. The functional interaction between these two transcription factors is further extended by the observation that STAT3 can specifically bind to HIF-1α target genes promoters, allowing the formation of transcriptionally active HIF-1α/RNA Polymerase II complexes [23]. Both these activities can contribute to the reported ability of constitutively active STAT3 to act as a first hit in oncogenic transformation [24]. Finally the pyruvate kinase M2 isoform (PKM2), an essential regulator of aerobic glycolysis that can be induced by HIF-1α, was shown to be able to directly promote chronic STAT3 Y-P. Thus STAT3, HIF-1α and PKM2 appear to take part in a positive feedback loop responsible for supporting cell proliferation and survival [25,26].

Protein acetylation, a crucial post-translational modification that affects gene expression by modulating both chromatin structure and the activity of many transcription factors, is a key factor in regulating proliferation and energy metabolism in both normal and transformed cells [27]. STAT3 can be acetylated (Ac-STAT3) by the CBP/p300 histone acetyltransferase in response to cytokines and growth factors, favoring dimer stability, tyrosine phosphorylation and transcriptional activity [28]. Interestingly, STAT3 acetylation was shown to occur in the nucleus thanks to a complex with the cancer stem cell marker CD44 and p300, leading to the binding to the cyclin D1 promoter, thus triggering increased expression and cell proliferation [29]. Ac-STAT3 is also thought to favor tumorigenesis by forming a complex with DNA methyltransferase 1 (DNMT1), thus leading to DNA methylation and silencing of tumor suppressor genes [30]. Along with other histone deacetylases, the NAD-dependent silent information regulator protein (SIRT)1 has been implicated in STAT3 deacetylation, in turn leading to reduced Y phosphorylation and transcriptional activity [31]. Recently, SIRT1 was also shown to indirectly modulate STAT3 S-P and its mitochondrial functions, since *Sirt1*-null MEF cells display high S-P STAT3 levels in mitochondria and increased mitochondrial activity [32]. Another SIRT family member, SIRT3, which localizes specifically to mitochondria, is implicated in down-regulating mitochondrial respiration. *Sirt3* genetic ablation leads to a metabolic switch towards aerobic glycolysis due to increased ROS production and consequent HIF-1α stabilization [33]. No direct interactions between SIRT3 and STAT3 have however been reported so far. Another player may be the mTORC1 inhibitor REDD1, a known HIF-1α target that localizes to mitochondria. REDD1 inactivation results in increased ROS production, stabilization of HIF-1α and tumorigenesis [34]. These observations suggest that SIRT3 and REDD1 might cooperate in mitochondria to sustain oxidative phosphorylation and ROS removal. Whether their activities may somehow regulate the functions of mitochondrial STAT3 remains to be established. On the other hand, the nutrient-sensing mTOR pathway has been implicated in regulating STAT3 activities by enhancing both its Y- and S-phosphorylation [35,36,37].

Interestingly, STAT3 was recently shown to play a role in the regulation of the autophagic pathway, another important metabolic process that has been implicated both positively and negatively in tumorigenesis [38]. This occurs in the cytoplasm, where non-phosphorylated STAT3 can interact via its SH2 domain with the autophagy inducer PKR/EIF2AK2 kinase, inhibiting its activity [39]. This function can be inhibited, in addition to interfering with the SH2 domain by means of specific compounds, by the activation of STAT3 Y-P, which decreases the pool of cytoplasmic STAT3 available for the interaction with PKR.

STAT3 appears to function as a hub to integrate different pro-survival and growth signals at the level of the energy and respiratory metabolism, via nuclear, mitochondrial and cytoplasmic activities mediated by its differentially modified forms [16]. Here, we discuss some of these results and how they may fit together in a multidimensional vision of STAT3 functions under conditions of aberrant activation.

## 2. Results and Discussion

### 2.1. STAT3 Constitutive Activation Elicits Pre-Oncogenic Features in Stat3^C/C^ MEFs.

**Figure 1:**
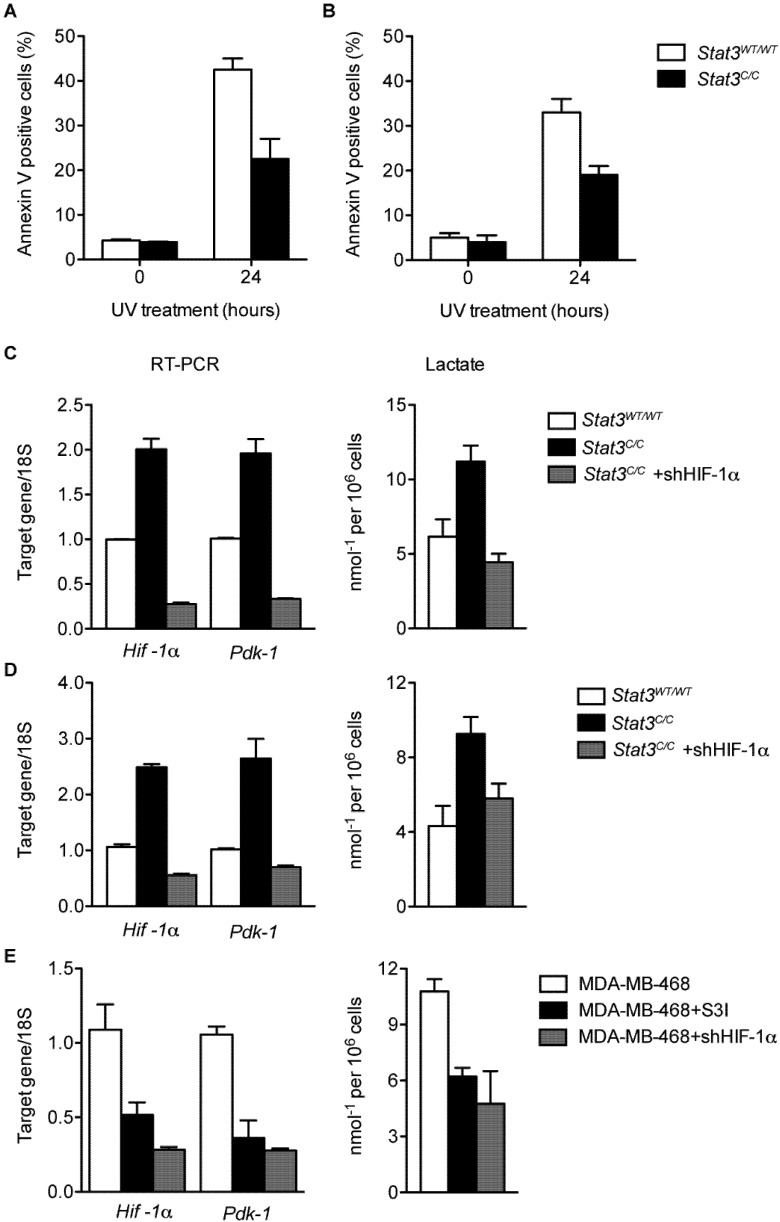
Primary (**A**) and immortalized (**B**) *Stat3^C/C^* MEFs are protected from apoptotic stimuli. *Stat3^WT/WT^* or *Stat3^C/C^* cells were treated with UV-B (10 J/m^2^) and after 24 h analyzed by flow cytometry for quantification of Annexin V positive cells. Aerobic glycolysis of *Stat3^WT/WT^* and *Stat3^C/C^* primary (**C**) and immortalized (**D**) MEFs was assessed by Taqman RT-PCR for *Hif*-1α and *Pdk*-1 mRNA levels and lactate production. Gene expression and lactate accumulation were also analyzed in MDA-MB-468 tumor cells (**E**), either untreated or treated with S3I (STAT3 inhibitor). All the above parameters were also measured upon *Hif*-1α silencing (sh*Hif*-1α). Bars represent mean values ± SEM, of three independent experiments. Modified from references [22,24], according to the journals’ license terms.

Mice homozygous for the *Stat3C* k/in allele exhibited increased nuclear localization, prolonged Y-P and enhanced transcriptional activity in response to cytokine stimuli in several tissues as well as in mouse embryonic fibroblasts (MEFs) [40]. Compared to wild-type control cells, *Stat3^C/C^* cells displayed enhanced proliferation and accelerated cell cycle, with a more rapid transition through the S-phase [22,24]. In addition, replicative senescence was strongly delayed and cells were highly resistant to apoptosis induced by different stimuli such as UV treatment (23% of apoptotic cells after 24 h, compared to 45% in the wild type controls, Figure 1A), H_2_O_2_ treatment and serum starvation ([22] and not shown). Interestingly, *Stat3^C/C^* MEFs became more readily immortalized than their wild type counterparts when subjected to the 3T3 spontaneous immortalization protocol (EF), and maintained significant resistance to multiple apoptotic stimuli, similar to the primary cells (19% of dead cells upon UV light exposure compared to 33% in wild type cells, Figure 1B) [24].

These features reflect the known activities of STAT3, promoting cell survival and proliferation, and suggest that low levels of constitutively active STAT3 can promote pre-oncogenic features in primary MEF cells.

### 2.2. Constitutive STAT3 Activity Induces a Metabolic Switch to Aerobic Glycolysis in MEFs and Tumor Cell Lines

A comparison of gene expression profiles in primary *Stat3^C/C^* and *Stat3^WT/WT^* MEFs performed via microarray analysis showed more than one thousand differentially expressed genes [22]. Gene Ontology (GO) analysis of up-regulated mRNAs revealed the presence of several genes involved in aerobic glycolysis including hypoxia inducible factor (*Hif*)-1α (Figure 1C), which is a known STAT3 transcriptional target [41]. The up-regulation of several known HIF-1α target genes, including pyruvate dehydrogenase kinase (*Pdk*)-1, the glucose transporter *Glut1* and several enzymes involved in glycolysis was also validated (Figure 1C and data not shown). These alterations correlated with enhanced production of lactate and increased intake of glucose (Figure 1C and data not shown). Thus, *Stat3^C/C^* MEFs appear to display a phenotype highly reminiscent of the well-known Warburg effect detected in most cancer cells, with glucose metabolism occurring mainly via aerobic glycolysis. These features were maintained in the immortalized *Stat3^C/C^* cells [24], which displayed STAT3-dependent increased levels of *Hif*-1α mRNA and of several of its target genes as well as higher lactate production (Figure 1D). These data demonstrated that low levels of aberrantly continuous STAT3 activation are sufficient to drive a metabolic switch towards aerobic glycolysis, which in turn is known to promote the ability of cancer cells to rapidly proliferate and to drive their plasticity to adapt and survive in environments of limiting oxygen concentrations. This novel function may explain the addiction for STAT3 shown by so many biologically different tumors. In order to verify these conclusions in a more physiological context with non mutant STAT3, we analyzed the expression of *Hif*-1α, *Pdk*-1 and other glycolytic genes in STAT3-dependent tumor cell lines, in the presence or absence of the S3I.201 STAT3 inhibitor. In agreement with the idea of STAT3 promoting aerobic glycolysis, all three cell lines tested, *i.e.*, MDA-MB-468, SKBR3 and DU145, displayed high expression of the *Hif*-1α and *Pdk*-1 mRNAs and high lactate production, which could be significantly down-regulated by STAT3 inhibition (Figure 1E and data not shown, [22]).

**Figure 2:**
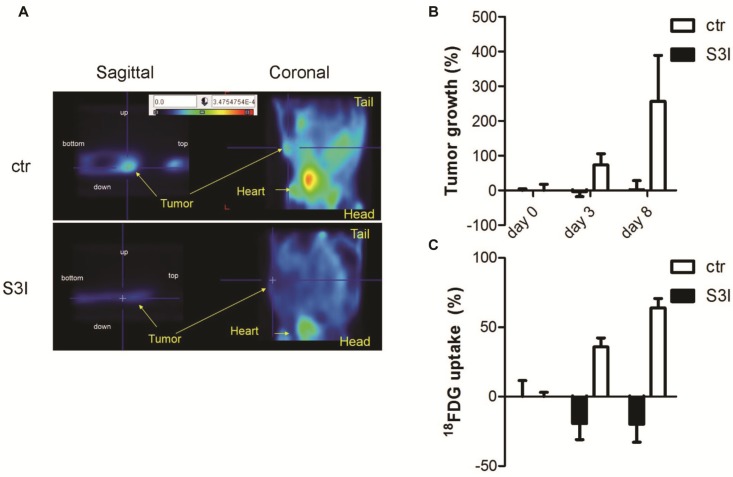
*In vivo* STAT3-dependent glycolysis of MDA-MB-468 tumor cells, measured by PET analysis as ^18^F-FDG uptake. Mice were inoculated with MDA-MB-468 cells and tumors let grow up to 60 mm^3^ prior to S3I treatment (day 0). ^18^F-FDG was injected and images acquired at days 0, 3 and 8 after the first S3I treatment. (**A**) Representative images (sagittal and coronal sections) of one control and one S3I-treated mouse 8 days after treatment; (**B**) Tumor volumes were measured with a caliper and indicated as percentage of growth relative to day 0; (**C**) The lower graph represents the variation of glucose uptake normalized over tumor volume at the indicated times after the first S3I treatment. Quantitative image analysis of tracer uptake was evaluated by drawing region of interest (ROI) of tumor on the transaxial images. Note decreased glucose uptake at day 3 (d3) and 8 (d8) upon S3I treatment, compared to constant tumor volume. % of ^18^F-FDG uptake = (SUV_d=n_ − SUV_d=0_) × 100/ SUV_d=0_.

The relevance of this novel metabolic STAT3 function in supporting the viability of tumor cells was also confirmed *in vivo* on xenograft tumors of MDA-MB468 cells. Since the levels of glucose uptake are considered a direct indication of glycolytic metabolism, we assessed them by means of PET analysis with the radioactive glucose-analogue ^18^F-FDG [22] (Figure 2). The tumors of control mice, not treated with the S3I compound, displayed regular growth and progressively increasing ratio between glucose uptake and tumor volume. Thus, glycolysis levels tended to increase even faster than tumor volume, indicating progressively enhanced Warburg metabolism. In contrast, treatment with the S3I inhibitor determined a growth arrest of the tumor, which correlated with a reduction in glucose uptake already after 3 days. These observations suggest that the inhibition of STAT3 activity has prominent effects on glucose metabolism also *in vivo*, and that this represents an important part of its pro-oncogenic activities.

Transcriptional induction is normally not believed sufficient to enhance HIF-1α expression at the protein level, due to its continuous proteasome-mediated degradation occurring in the absence of specific signals such as hypoxia or growth factors stimulation [42]. However, we could show that *Stat3^C/C^* MEFs display higher HIF-1α protein expression than their wild type controls [22]. This suggests that continuous, low level mRNA induction triggered by constitutively active STAT3 can lead to a small (50%) increase in HIF-1α protein levels that is sufficient to enhance protein activity. In turn, enhanced HIF-1α activity could explain the STAT3-dependent metabolic switch towards glycolysis described above. Indeed, shRNA-mediated silencing of *Hif*-1α completely normalized glycolysis levels in both primary and immortalized *Stat3^C/C^* MEFs, leading to down-regulation of many genes involved in aerobic glycolysis including *Pdk*-1, and to reduction of lactate production, glucose intake and sensitivity to glucose deprivation (Figure 1C,D and [22,24]). *Hif*-1α silencing could also down-regulate *Pdk*-1 expression and lactate production in STAT3-addicted tumor cells, to an extent similar to that obtained by STAT3 silencing (Figure 1E). Thus, STAT3-dependent *Hif*-1α induction appears to represent the main mechanism responsible for the increased glycolysis and enhanced glucose dependence observed in the *Stat3^C/C^* MEFs, strongly contributing to the *in vivo* growth of MDA-MB-468 tumor xenografts. Of note, increased HIF-1α activity may enhance PKM2 expression and initiate the positive feedback loop leading to continuous STAT3 Y-P [25].

Despite a well-accepted pro-tumorigenic role, STAT3 activity has also been correlated with good prognosis in specific tumors [43,44] and mouse models of colorectal [45] and thyroid cancer [46]. In particular in the latter, STAT3 Y-P negatively correlates with tumor size and aggressiveness in human papillary thyroid carcinomas, and appears to paradoxically negatively regulate *Hif*-1α expression and aerobic glycolysis under hypoxic conditions [46]. These observations suggest that the specific role of STA3 can be strongly tissue- and context-dependent.

### 2.3. Reduced Mitochondrial Activity in Cells with a Constitutive Activation of STAT3

**Figure 3:**
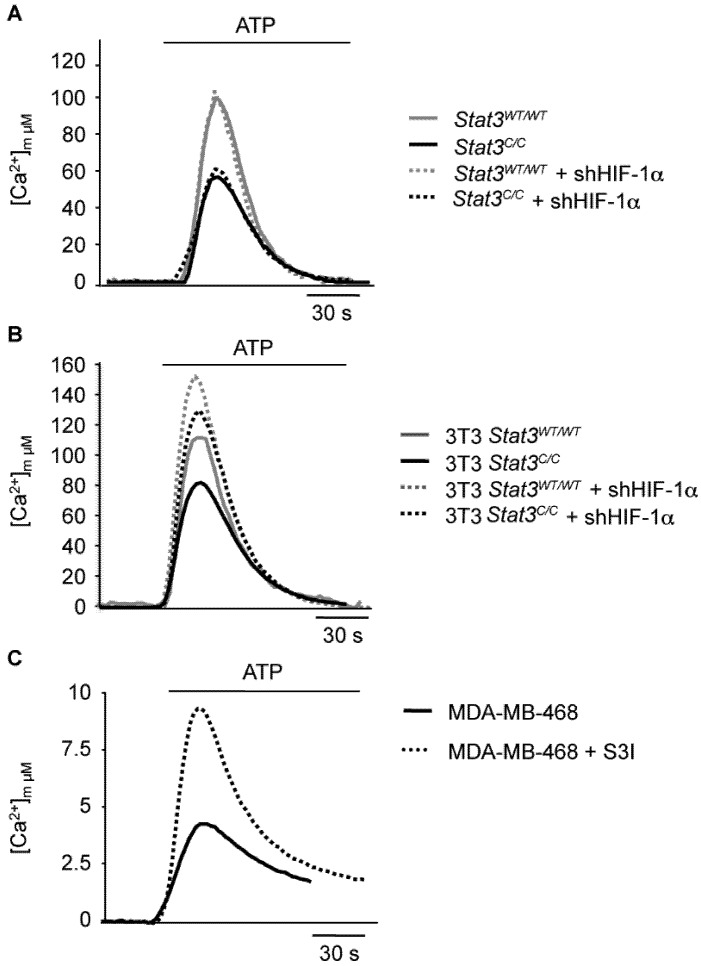
Decreased mitochondrial activity of primary (**A**) or immortalized (**B**) *Stat3^C/C^* MEFs, and of MDA-MB-468 tumor cells (**C**). MEFs of the indicated genotypes, either silenced or not for *Hif*-1α, were transduced with a mitochondrial-targeted aequorin (AEQ) [55]. Aequorin activity was measured upon challenging with 100 μM ATP as indicated. Mitochondrial Ca^2+^ response was assessed in MDA-MB-468 cells treated or not with S3I for 12 h (**C**). Data are representative of at least 10 traces, each from three independent experiments. Modified from references [22,24], according to the journals’ license terms.

In addition to enhanced aerobic glycolysis, tumor cells that have undergone the Warburg effect also display decreased oxidative respiration in mitochondria, which is partly the consequence of deviating pyruvate towards glycolysis and may contribute to down-regulate ROS production and counteract senescence [47]. Interestingly, in parallel to the up-regulation of genes involved in glycolysis, microarray analysis of *Stat3^C/C^* and *Stat3^WT/WT^* MEFs showed significant down-regulation of genes belonging to GO categories related to mitochondrial function [22]. In particular, the expression of nuclear-encoded genes involved in mitochondrial function was significantly reduced, correlating with reduced protein levels of representative components of the Electron Transport Chain (ETC). This down-regulation may lead to reduced cellular respiration, similar to what observed in cancer cells displaying aerobic glycolysis and the Warburg effect. Mitochondrial activity was assessed as the measure of Ca^2+^ uptake upon ATP stimulation [48], which directly regulates oxidative phosphorylation [49,50,51,52]. Indeed, mitochondrial Ca^2+^ uptake was significantly reduced as compared to controls in primary (Figure 3A) as well as immortalized (Figure 3B) *Stat3^C/C^* MEFs. Accordingly, both mitochondrial ATP production and basal respiratory chain activity were reduced [22]. Despite this observation, the ATP:ADP ratio was increased in the *Stat3^C/C^* MEFs, confirming that these cells rely on energy production generated via glycolysis [22]. Similar to MEFs, also the STAT3-addicted MDA-MB-468, SKBR3 and DU145 human tumor cells exhibited relatively low mitochondrial activity that was dependent on STAT3, since it could be enhanced by treatment with the S3I inhibitor (Figure 3C and [22]). Interestingly, a reduced mitochondrial Ca^2+^ uptake dramatically blunts the apoptotic response [53] preventing the mitochondrial permeability transition pore opening [54], as observed in *Stat3^C/C^* cells [22].

Importantly, and in contrast to what observed for glycolysis, the reduced mitochondrial activity observed in primary MEFs was independent from HIF activity, as shown by the failure of *Hif*-1α silencing to increase Ca^2+^ uptake (Figure 3A and [22]). One possible explanation is the observed STAT3-dependent down-regulation of nuclear genes encoding for mitochondrial proteins and the consequent reduced levels of ETC components. Accordingly, *Hif*-1α silencing could not rescue the expression of these genes [22]. Mitochondrial activity was independent of HIF-1α levels also in the MDA-MB-468 cells (not shown).

In contrast to primary cells, in immortalized MEFs *Hif*-1α silencing could enhance Ca^2+^ uptake extent in both wild type and *Stat3^C/C^* cells (Figure 3B), suggesting that its activity contributes to the control of oxidative phosphorylation in immortalized fibroblasts regardless of their genotype [24]. However, the levels of Ca^2+^ uptake remained significantly lower in *Stat3^C/C^* cells as compared to the wild type controls even after *Hif*-1α silencing. Thus, the control of mitochondrial activity in these cells remains at least partly HIF-independent also in immortalized cells.

**Figure 4:**
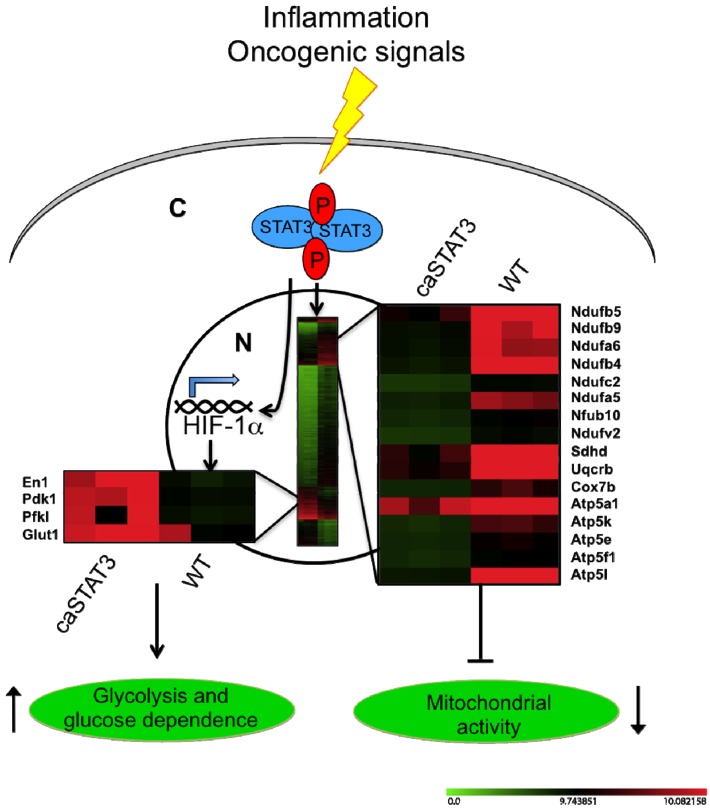
Constitutively active STAT3 regulates mRNA expression of metabolic genes. Y-P STAT3 is the downstream effector of several oncogenic signals. Constitutively activated STAT3 translocates into the nucleus where it induces aerobic glycolysis through (i) the up-regulation of HIF-1α, which in turn mediates the induction of several mRNA involved in glycolysis; and (ii) the down-regulation of mitochondrial genes and of ETC-complexes, leading to reduced mitochondrial activity. The heatmaps display the mean log2 fold change of signal intensity between *Stat3^C/C^* and wild type MEFs as assessed by microarray analysis.

Taken together, these data suggest that constitutively active STAT3, observed downstream of continuous stimulation by inflammatory cytokines such as IL-6 and oncogenes, promotes a Warburg-like metabolic switch via two distinct nuclear mechanisms (Figure 4). First, the induction of *Hif*-1α expression, which in turn mediates the up-regulation of genes involved in glycolysis. This increases glucose consumption and promotes fast proliferation, leading to glucose dependence like in glycolytic cancer cells. Second, the down-regulation of mitochondrial activity, which is totally or partly HIF-1α-independent and may be caused by the reduced levels of ETC components, in turn caused by reduced expression of nuclear genes encoding for mitochondrial proteins. Although at present we cannot determine whether STAT3 can directly affect their transcription, we did not observe any enrichment in STAT3 responsive elements in the promoters of the down-regulated genes [56]. Thus, we favor the idea of an indirect regulation of a common repressor or of targeting microRNA(s). Impaired mitochondrial activity may contribute to the reduced production of ROS observed in the *Stat3^C/C^* MEFs, which in turn is likely correlated to their high resistance to apoptosis and senescence, two hallmarks of cellular transformation. On the other hand, STAT3 may help regulating energy metabolism also via different mechanisms. For example, it has been reported that human and murine hepatocellular carcinomas show significantly reduced expression of gluconeogenic enzymes including PCG1α, mediated by the up-regulation of miR-23a expression by an activated IL-6-STAT3 signalling pathway [57]. This leads to increased glucose release, thus sustaining fast proliferation. On the other hand, PCG1α is known to affect the levels and activity of numerous mitochondrial proteins, positively regulating mitochondrial respiration [58,59], and to control ROS levels by regulating the expression of numerous ROS-detoxifying enzymes [60]. Thus, STAT3-dependent down-regulation of PCG1α may also contribute to down-regulate the levels of ETC components as well as mitochondrial activity.

Despite the decreased expression of about 500 mitochondrial genes and the normal mitochondrial morphology [22], the mitochondrial mass was increased in the *Stat3^C/C^* MEFs [61], suggesting a potential role of STAT3 in mitochondrial biogenesis. This might occur through the transcriptional induction of c-myc, a *bona fide* STAT3 transcriptional target whose levels are increased in the *Stat3^C/C^* MEFs and that is a well-known inducer of mitochondrial biogenesis [62]. 

### 2.4. STAT3C Triggers Tumorigenic Transformation in Immortalized MEFs

**Figure 5:**
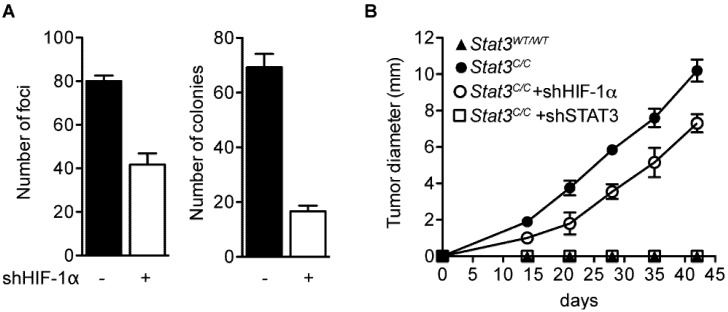
Immortalized *Stat3^C/C^* MEFs were silenced (white bars) or not (black bars) for *Hif*-1α and their ability to form foci and colonies in soft agar was measured (**A**). Immortalized MEFs of the indicated genotypes and treatments were tested for their ability to grow *in vivo* in nude mice (**B**). Bars represent mean values ± SEM of three independent experiments. Modified from reference [24], according to the journal’s license terms.

Similar to primary cells, spontaneously immortalized *Stat3^C/C^* MEFs proliferated much faster than their wild type counterparts, and displayed loss of inhibition contact with a tendency to grow in multi-layers [24], a feature typical of transformed cells, leading us to assess other transformation phenotypes such as focus forming ability and anchorage-independent growth. In contrast to their wild type controls, immortalized *Stat3^C/C^* MEFs were able to give rise to foci on plastic and to colonies in soft agar. HIF activity was only partially responsible for these features, which could not be completely reverted by *Hif*-1α silencing (Figure 5A and [24]). Finally, immortal *Stat3^C/C^* MEFs were able to give rise to tumors in nude mice, confirming full malignant transformation. In vivo growth was totally STAT3-dependent and only in part mediated by HIF-1α, as shown by silencing experiments (Figure 5B and [24]).

In contrast to primary MEFs, which require two oncogenic hits to become transformed, MEFs spontaneously immortalized via the 3T3 protocol [63] are known to become competent for transformation elicited by a single oncogene [64], suggesting that they have already undergone one oncogenic hit. Our results show that low levels of constitutively active STAT3 are sufficient to transform primary cells to full malignancy upon immortalization via a 3T3 protocol, suggesting therefore that chronic STAT3 activity can act as a first hit in multistep carcinogenesis. This observation might explain the key role of STAT3 in the causative link between chronic inflammation and cancer, since IL-6-driven STAT3 constitutive activity is a hallmark of chronic inflammation [65]. According to our data, cells exposed to chronic IL-6 signalling and displaying continuous STAT3 activation behave like cells that have undergone a first oncogenic mutation, and will therefore be exquisitely sensitive to mutagenic events occurring in the inflammatory microenvironment, rich in cytokines, growth factors and reactive oxygen species.

Thus, there are many mechanism(s) through which constitutively active STAT3 sensitizes cells to tumorigenic transformation, belonging to classical well described functions as well as to the novel metabolic role that we have recently described. A prominent feature is no doubt played by the resistance to programmed cell death in response to many different apoptotic stimuli. This is a well-known function of STAT3, triggered also in our system by the transcriptional induction of anti-apoptotic genes as well as by the reduced mitochondrial Ca^2+^ uptake [22,24]. The delayed senescence correlating with decreased ROS production [22] and the increased proliferation here described likely correlate with the metabolic switch towards aerobic glycolysis. Aerobic glycolysis is known to allow rapidly proliferating cells to accumulate NADPH and carbon skeletons needed for anabolic reactions as a side-product of glucose consumption and ATP production [15]. Additionally, decreased mitochondrial activity will help switching the energy balance towards glycolysis allowing at the same time to reduce ROS production, which will in turn help cell survival. Another important player in oncogenesis is c-myc, which contributes to both increased proliferation and aerobic glycolysis and is a well known STAT3 target. Indeed the levels of c-myc are elevated in the *Stat3^C/C^* MEFs [66]. Finally, *Hif*-1α induction may initiate the already mentioned feed forward loop involving PKM2 and STAT3 that would contribute maintaining high HIF-1α activity, high PKM2:PKM1 ratios and constitutive STAT3 Y-P [26].

## 3. Conclusions

**Figure 6:**
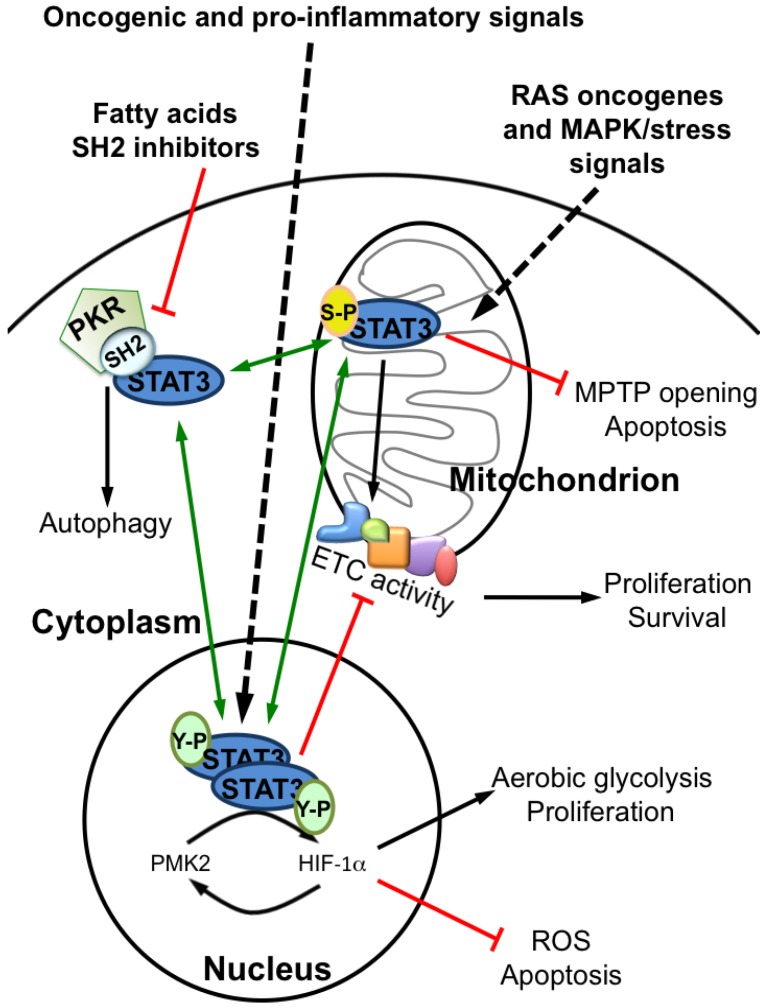
STAT3 can be localized in the mitochondrial matrix, in the nucleus and in the cytoplasm, according to specific post-transcriptional modifications triggered by different upstream stimuli. Many oncogenic and inflammatory signals mainly induce Y-P STAT3 nuclear functions that, in addition to known canonical functions, mediate the induction of *Hif*-1α and lead to increased glycolysis and to the initiation of a positive feedback between HIF-1α, PKM2 and STAT3. This contributes to supporting cell proliferation and protection from apoptosis and senescence. On the other hand, nuclear Y-P STAT3 reduces mitochondrial activity by down-regulating, directly or indirectly, many ETC complexes mRNAs, leading to reduced mitochondrial respiration, ROS production and apoptosis. In contrast, RAS oncogenic signals and MAP kinases trigger S-P and mitochondrial STAT3 functions, improving cell survival by enhancing both oxidative phosphorylation and aerobic glycolysis, and protecting cells from apoptosis by inhibiting the opening of the mitochondrial permeability transition pore (MPTP). Finally, cytoplasmic STAT3 can inhibit autophagy by interacting with the cytoplasmic Protein Kinase R (PKR) via its SH2 domain. The relative abundance of each form of STAT3 is the result of a balance with the other forms, in turn determined by the interplay of different activating and inactivating signals. Black arrows: induction; green arrows: interplay; red lines: inhibition.

STAT3 constitutive activation is featured by many types of tumors, and mainly occurs downstream of the activation of several different oncogenic pathways [11,67]. Accordingly, STAT3 is required for malignant transformation by many different oncogenes that trigger STAT3 Y-P, the prototype being vSrc, in part by acting on glucose metabolism as described here. On the other hand, also oncogenes of the RAS family have been shown to require STAT3 to transform cells, and this occurs via phosphorylation on serine rather than on tyrosine [17,68]. Also S-P STAT3 mediates important metabolic functions when localized to the mitochondrion, including preservation of mitochondrial activity, induction of aerobic glycolysis and inhibition of the opening of the mitochondrial permeability transition pore. Thus, STAT3 appears to be able to promote aerobic glycolysis both from within the nucleus and the mitochondrion, while it plays opposite roles on mitochondrial activity, depending on the oncogenic signal and the mode of activation (*i.e.*, Y- *versus* S-P). To further complicate the picture, also cytoplasmic, non-phosphorylated STAT3 is implicated in regulating cell survival and energy metabolism through autophagy inhibition and, finally, STAT3 acetylation/deacetylation status, regulated by oncogenes, transcriptional co-activators and different classes of HDAC enzymes, can affect the activities of both Y-P and S-P STAT3. These observations contribute to explain the multiform and sometimes contrasting activities described for STAT3, since its functions will vary according to cell type-specific target genes, mode of activation and sub-cellular localization. In turn, any condition altering the cellular concentration of one specific form of STAT3 is bound to affect the functions of all other forms by directly or indirectly altering their abundance, as depicted in Figure 6. This consideration needs to be kept in account when designing STAT3-inhibiting drugs.

A deeper understanding of the interplay between the differentially phosphorylated forms of STAT3 and their relative sub-cellular distribution under specific pathological conditions and in different tumor types may help designing function-specific inhibitors that may be tested as targeted therapeutic approaches.
